# Supplementary material for: FERN – a Java framework for stochastic simulation and evaluation of reaction networks
Source: BMC Bioinformatics. 2008 Aug 29;9:356. doi: 10.1186/1471-2105-9-356 (PMC2553347; doi:10.1186/1471-2105-9-356)
Supplement: Additional file 1 — FERN distribution, Version 1.3. This archive contains the FERN source code and binaries as well as documentation and example models in FernML and SBML. [file 1471-2105-9-356-S1.zip › fern/doc/javadoc/fern/simulation/observer/ReactionIntervalObserver.html]

ReactionIntervalObserver


---


|  |  |  |
| --- | --- | --- |
| **Overview** \| **Package** \| **Class** \| **Use** \| **Tree** \| **Deprecated** \| **Index** \| **Help** | |  |
| **PREV CLASS**   **NEXT CLASS** | **FRAMES**    **NO FRAMES**     **All Classes** |  |
| SUMMARY: NESTED \| FIELD \| CONSTR \| METHOD | DETAIL: FIELD \| CONSTR \| METHOD |  |


---


## fern.simulation.observer Class ReactionIntervalObserver

```
java.lang.Object
  fern.simulation.observer.Observer
      fern.simulation.observer.IntervalObserver
          fern.simulation.observer.ReactionIntervalObserver
```

**All Implemented Interfaces:**: GnuPlotObserver

---

``` public class ReactionIntervalObserver extends IntervalObserver ```

Observes number of firings of reactions repeatedly after certain intervals.

This observer does take repeats into account. If you repeat the simulation,
you will get an average over the results of each run.

**Author:**
:   Florian Erhard

---

**Constructor Summary**

`ReactionIntervalObserver(Simulator sim,
double interval,
int... reactions)`
  
          Creates the observer for a given simulator, a given interval and given reaction indices.

`ReactionIntervalObserver(Simulator sim,
double interval,
String... speciesNames)`
  
          Creates the observer for a given simulator, a given interval and given neighbors.


| **Method Summary** | |
| --- | --- |
| `void` | `activateReaction(int mu, double t, Simulator.FireType fireType, int times)`             Record the firing of mu, if mu is registered. |
| `protected  double` | `getEntityValue(int i)`             Gets the actual number of firings of a reaction since the simulation started. |
| `double` | `getRecordStart()`             Gets the moment in time where the recording starts |
| `String[]` | `getStyles()`             Gets the styles for the columns. |
| `void` | `setRecordStart(double recordStart)`             Sets the moment in time where the recording starts |
| `void` | `started()`             Clears the recorded values and registers (if needed) `theta` |

**Methods inherited from class fern.simulation.observer.IntervalObserver**

`finished, getFinalValue, getRecentData, isPlotQuality, isThetaMethod, setPlotQuality, setThetaMethod, step, theta, toGnuplot, toGnuplot, toGnuplotRecent, toGnuplotRecent, toString`

**Methods inherited from class fern.simulation.observer.Observer**

`applyLabelFormat, getLabel, getLabelFormat, getNumSimulations, getPrintWriter, getSimulator, getTheta, getTitlesCommand, print, setLabelFormat, setPrintWriter, setSimulator, setTheta`

**Methods inherited from class java.lang.Object**

`clone, equals, finalize, getClass, hashCode, notify, notifyAll, wait, wait, wait`

**Constructor Detail**

### ReactionIntervalObserver

```
public ReactionIntervalObserver(Simulator sim,
                                double interval,
                                String... speciesNames)
```

:   Creates the observer for a given simulator, a given interval and given neighbors.

    **Parameters:**: `sim` - simulator: `interval` - interval: `speciesNames` - the names of the species, each neighboring reaction of any of these is added

---


### ReactionIntervalObserver

```
public ReactionIntervalObserver(Simulator sim,
                                double interval,
                                int... reactions)
```

:   Creates the observer for a given simulator, a given interval and given reaction indices.

    **Parameters:**: `sim` - simulator: `interval` - interval: `reactions` - reaction indices


**Method Detail**

### activateReaction

```
public void activateReaction(int mu,
                             double t,
                             Simulator.FireType fireType,
                             int times)
```

:   Record the firing of mu, if mu is registered.

    :   **Overrides:**: `activateReaction` in class `IntervalObserver`
    :   **Parameters:**: `mu` - the reaction which is supposed to fire: `t` - the time the reaction fires (at this time `Simulator.getTime()` does not necessarily yield the firing time): `fireType` - the type of the firing: `times` - TODO

---


### started

```
public void started()
```

:   **Description copied from class: `IntervalObserver`**
:   Clears the recorded values and registers (if needed) `theta`

    :   **Overrides:**: `started` in class `IntervalObserver`

---


### getRecordStart

```
public double getRecordStart()
```

:   Gets the moment in time where the recording starts

    :   **Returns:**: the recordStart

---


### setRecordStart

```
public void setRecordStart(double recordStart)
```

:   Sets the moment in time where the recording starts

    :   **Parameters:**: `recordStart` - the recordStart to set

---


### getEntityValue

```
protected double getEntityValue(int i)
```

:   Gets the actual number of firings of a reaction since the simulation started.

    :   **Specified by:**: `getEntityValue` in class `IntervalObserver`
    :   **Parameters:**: `i` - index of the entity **Returns:**: value of the entity

---


### getStyles

```
public String[] getStyles()
```

:   **Description copied from interface: `GnuPlotObserver`**
:   Gets the styles for the columns. If you don't want styles, just return null!

    :   **Returns:**: styles for the columns


---


|  |  |  |
| --- | --- | --- |
| **Overview** \| **Package** \| **Class** \| **Use** \| **Tree** \| **Deprecated** \| **Index** \| **Help** | |  |
| **PREV CLASS**   **NEXT CLASS** | **FRAMES**    **NO FRAMES**     **All Classes** |  |
| SUMMARY: NESTED \| FIELD \| CONSTR \| METHOD | DETAIL: FIELD \| CONSTR \| METHOD |  |


---
